# Halide perovskites scintillators: unique promise and current limitations†

**DOI:** 10.1039/d1tc01595h

**Published:** 2021-06-01

**Authors:** Oliver D. I. Moseley, Tiarnan A. S. Doherty, Richard Parmee, Miguel Anaya, Samuel D. Stranks

**Affiliations:** Cavendish Laboratory, University of Cambridge, JJ Thomson Avenue Cambridge CB3 0HE UK sds65@cam.ac.uk ma811@cam.ac.uk; Cheyney Design and Development, Ltd., Litlington Cambridge SG8 0SS UK; Department of Chemical Engineering & Biotechnology, University of Cambridge, Philippa Fawcett Drive Cambridge CB3 0AS UK

## Abstract

The widespread use of X- and gamma-rays in a range of sectors including healthcare, security and industrial screening is underpinned by the efficient detection of the ionising radiation. Such detector applications are dominated by indirect detectors in which a scintillating material is combined with a photodetector. Halide perovskites have recently emerged as an interesting class of semiconductors, showing enormous promise in optoelectronic applications including solar cells, light-emitting diodes and photodetectors. Here, we discuss how the same superior semiconducting properties that have catalysed their rapid development in these optoelectronic devices, including high photon attenuation and fast and efficient emission properties, also make them promising scintillator materials. By outlining the key mechanisms of their operation as scintillators, we show why reports of remarkable performance have already emerged, and describe how further learning from other optoelectronic devices will propel forward their applications as scintillators. Finally, we outline where these materials can make the greatest impact in detector applications by maximally exploiting their unique properties, leading to dramatic improvements in existing detection systems or introducing entirely new functionality.

## Abstract 2

The origins of the remarkable performance of halide perovskite scintillators are presented, along with solutions to the challenges facing the field, followed by a discussion of applications that will benefit from the unique properties of these materials.

## Graphical abstract

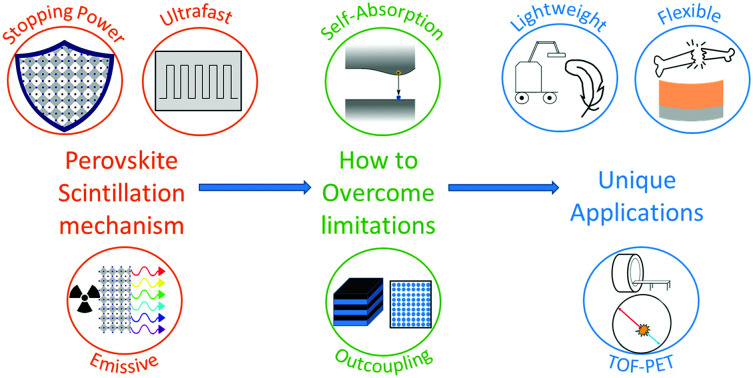

## 1. Introduction

**Fig. 1:**
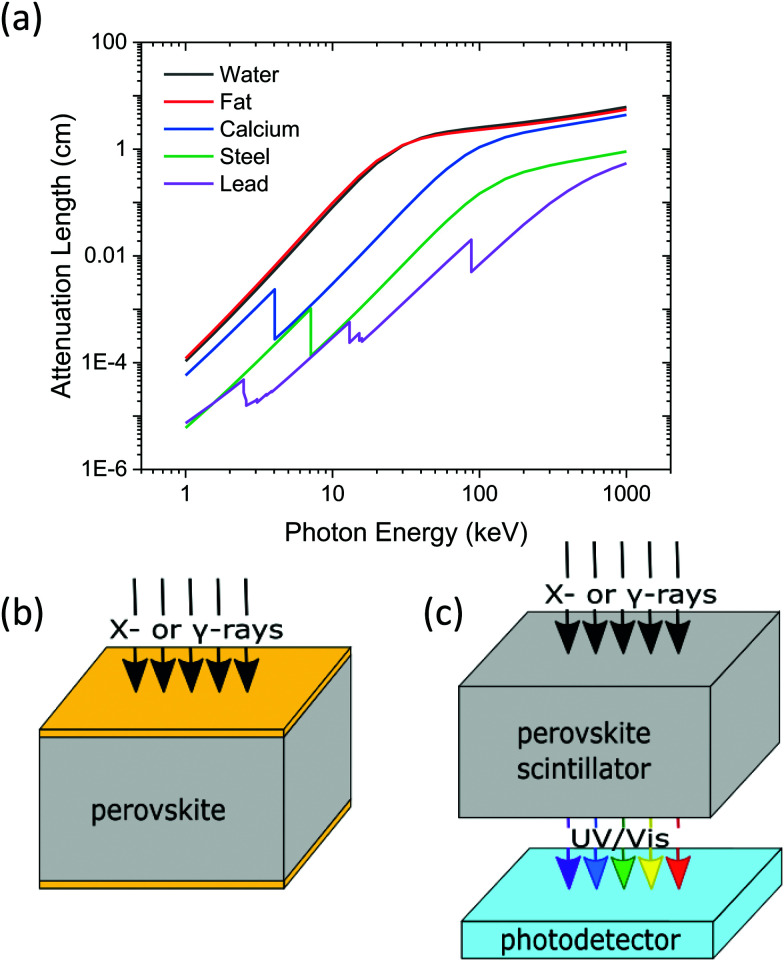
(a) The strong increase in absorption length as photon energy increases for a range of materials. The fat (red) component represents a common triglyceride molecule common in animal fat cells. The difference in absorption between calcium in bones and organics in tissue enables contrast images to be generated. (b and c) Schematics of (b) direct and (c) indirect detection mechanisms. In the case of direct detectors, metal electrodes are employed to extract generated charge carriers to produce the signal. For indirect detectors, the incident beam impinges on the scintillator material and generates UV/Vis photons, which are then collected by a photodetector to produce the signal.

Ionising radiation in the form of X- and gamma-rays are powerful modern diagnostic tools. These energetic photons, typically kiloelectron volts and above, exhibit long penetration depths that exceed the millimetre scale^1^ (Fig. 1a) and enable a diverse range of applications, including healthcare,^2^ industrial screening,^3,4^ non-destructive imaging,^5^ spectroscopy,^6^ astronomy,^7,8^ and security.^9^ The usefulness of ionising radiation is dependent on the radiation source, the sample, the associated interface electronics, dedicated image processing algorithms and, crucially, the radiation detector itself.^10^ Solid-state radiation detection can be split into two distinct categories, direct and indirect, which differ in the way they generate a signal from the incident radiation (Fig. 1b and c). Direct detectors harvest the incident radiation and produce energised charge carriers, which are extracted by electrodes to generate a signal (Fig. 1b).^11^ Despite the relative simplicity of such devices, their commercial application is hindered by the constraints imposed by deeply penetrating radiation. Charge collection distances have to be on the order of the material thickness, and as the radiation energy increases above 40 keV and typical absorption depths exceed the millimeter scale, efficient and fast charge extraction becomes difficult. Consequently, direct detection is only employed for soft X-rays due to their shorter penetration depths, for example in mammography utilizing < 40 keV X-rays^2^ and medical device inspection.^12^

Indirect detectors involve a radiation-sensitive material that down-converts the incident beam into UV/Vis light, which is then collected by an array of sensitive photodetectors, such as PIN diodes or photomultiplier tubes. This emissive material is known as a scintillator, and the light produced from ionising radiation excitation is radioluminescence (RL). Given there are no long-range charge collection requirements, scintillators can retain detection properties when the material thickness exceeds millimeters, enabling use across the whole X- and gamma-ray spectrum. Furthermore, decoupling the detection process into two distinct steps permits each to be optimized separately, allowing decades of advancements in photodetector development to be exploited. Fast scintillator emission is suitable for high-speed applications^13^ and high imaging frame rates, with the sub-nanosecond RL decay times achievable far exceeding the charge collection times in direct detectors.^14,15^ As a result, indirect detectors dominate applications, from medical radiography to particle collider calorimeters. However, typical commercial scintillators, such as CsI:Tl, are hindered by their high production costs and fixed emission wavelengths, limiting their functionality with substrates and detectors. Furthermore, the diverse range of applications makes it near-impossible to produce a one-size-fits-all scintillator. Developing new detectors with increased functionality and performance is of great importance, especially in medical imaging, where the benefits of the procedure must be carefully balanced against the increased risk of cancer associated with patient radiation exposure.^16^ Whilst existing techniques, such as conventional transmission radiography, can provide useful diagnostic information at safe dose levels, more advanced techniques require the patients to be monitored to ensure that cumulative dose does not exceed safe limits. This is particularly important for the increasing use of computed tomography (CT, 5 million yearly scans in the UK alone^17^), with each scan administering up to 10 mSv (equivalent to 4.5 years of natural background radiation);^18^ low dose radiography will be very advantageous for such applications.

Recently, a unique class of semiconductors, halide perovskites (PVKs) have demonstrated a series of breakthroughs in a range of optoelectronic applications. Most notably, PVK solar cells have reached power conversion efficiencies of over 25% in little more than a decade,^19^ owing to strong direct band gap absorption, excellent charge transport properties and high defect tolerance.^20^ Together with these ideal optoelectronic properties, the high atomic weight of the constituent elements render PVKs promising candidates for ionising radiation detector materials. PVKs were demonstrated as a direct detector material following the synthesis of millimeter-sized single crystals, with X-ray-induced currents reported in CsPbBr_3_^21^ and MAPbI_3_^22^ (MA = methylammonium) crystals, and improvements in crystal quality^23^ and device integration^24^ advanced the field quickly. PVK direct detectors have achieved ultralow detection limits (<10 nGys^−1^)^25,26^ and up to 100 times the sensitivity of their a-Se counterpart^27^ for soft X-rays, making them exciting commercial prospects. However, high dark currents of 23 nA cm^−2^ ^27^ even under small external fields remain a problem for direct PVK detectors, in part due to the low bulk resistivity and the bias instabilities caused by ion migration. Growth methods,^28^ compositional engineering,^29^ passivation^30^ and dimensionality tuning^31^ are all promising solutions to these issues. The reader is referred to a comprehensive review of PVKs for direct detection.^32^

PVKs were first proposed as scintillators in the 1990s,^33–36^ an application revived in the last few years following great success of PVKs as light emitters with high photoluminescence quantum efficiencies and colour control from band gap and dimensionality tuning. Recently, scintillation has been demonstrated in a range of PVKs from bulk crystals^37^ to nanocrystals,^38,39^ and they remain an exciting prospect to offer sub-nanosecond emission with high light yields from a material that can be synthesised inexpensively and at low temperatures. The rapid advancement of PVK scintillators, already outperforming some commercial materials, combined with the dominance of indirect systems in practical detector applications has generated industrial interest in the materials.^40^ Herein, we dissect the operating mechanisms and advantages of PVKs in indirect detectors, highlighting areas where they can replace traditional materials, or offer unique properties that can be exploited. The limitations of PVKs are discussed, and pathways to further improve the materials are proposed, utilising ongoing knowledge derived from the concurrent development of other PVK optoelectronic devices. Finally, applications where PVKs may find unique commercial use are outlined.

## 2. General scintillator considerations

### 2.1 Mechanism

**Fig. 2:**
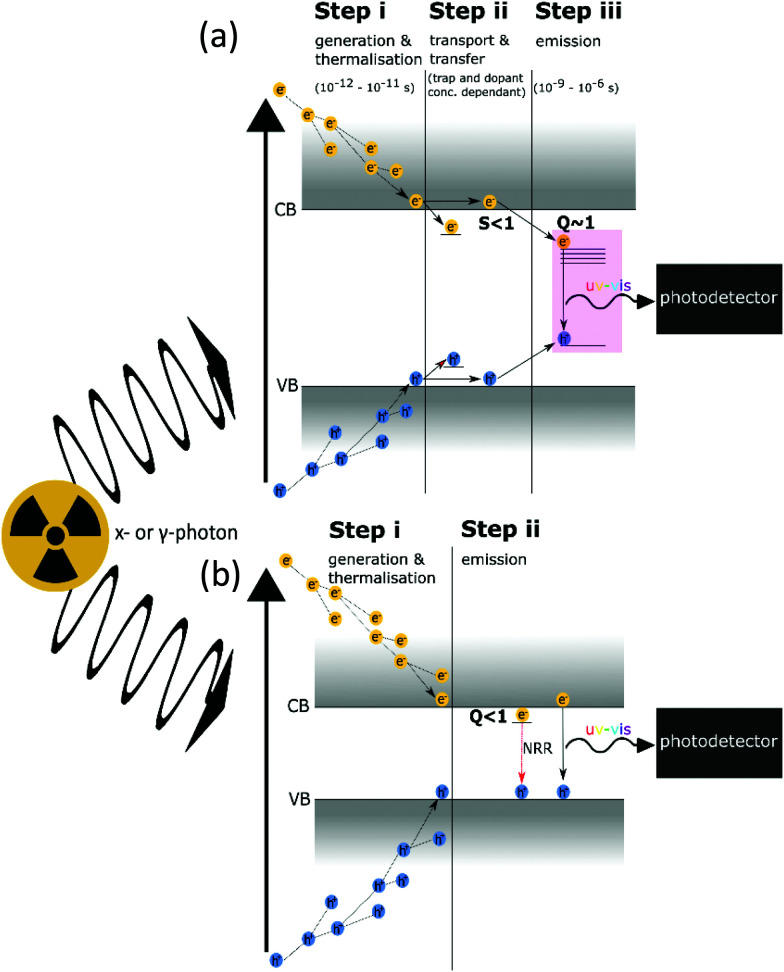
Schematic of the scintillation mechanism in extrinsic (a) and intrinsic (b) emitter systems (NRR = non-radiative recombination). The additional transfer step to the dopant energy levels (pink box) in extrinsic emitters increases the likelihood of charge carrier trapping causing losses and afterglow.

While the exact mechanisms of RL are complex and material-dependent, the general process can be simplified into three key steps: (i) generation and relaxation of excited charge carriers, (ii) transport of carriers to emission centres, and (iii) radiative recombination of carriers (Fig. 2). A prediction for the maximum number of photons emitted (*N*_ph_) is possible by looking at each of these steps in eqn (1):

1 *N*_ph_ = *B* × *S* × *Q*

2
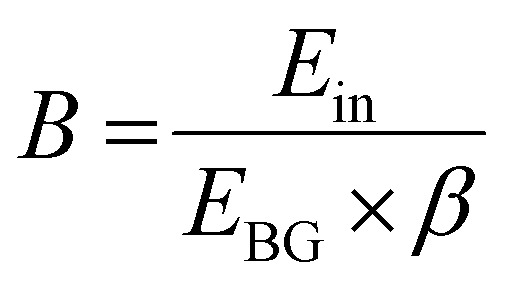
Here, *B* represents the number of carriers generated per incident photon (step i) and is a function of the incident photon energy, *E*_in_, the band gap, *E*_BG_, and a material-dependent *β* term. *S* and *Q* represent the quantum efficiencies of the carrier transfer (step ii) and radiative recombination (step iii) processes, respectively. Attenuation of high energy photons occurs by the photoelectric effect, Compton scattering and, for energies greater than twice the rest mass of electrons (*E* > 1.022 MeV), pair production. The probability of photoelectric absorption scales approximately as a function of *Z*^5^/*E*_in_^3.5^ and Compton scattering *Z*/*E*_in_, leading to the requirement of high atomic number constituents for efficient attenuation. The photoelectric effect generates a hot electron and hole, which cause a cascade of carrier generation *via* secondary X-rays, Auger events and further Compton scattering (timescale ∼1–100 fs). Once below the threshold for further ionization, carriers thermalise (timescale ∼1–10 ps) to the band edge.^41^ The *β* term is material-specific and can be predicted using existing models.^42–44^ The value of *β* is approximately 3 for semiconductors, whereas 2 < *β* < 3 for ionic crystals, leading to the generation of approximately 65 000 carriers per MeV photon in CsI:Tl.^45^ Carriers are then transferred to emission centres where they can emit in step iii.

A key mechanistic difference between different scintillator materials is the origin of the RL being intrinsic to the material, or the result of an extrinsic dopant. Extrinsic emitters, including CsI:Tl, GOS:Tb (Gd_2_O_2_S), and LYSO:Ce (Lu_2(1-x)_Y_2x_SiO_5_), are common commercially and the properties of extrinsic emission (Fig. 2a) depend on the activator ion.^46^ However, limitations of extrinsic materials arise in steps i and ii in the scintillation process. Firstly, to ensure efficient charge transfer in step ii, prevent thermal repopulation of emissive states, and reduce reabsorption of the scintillated light, the matrix band gap must be wider than the band gap of the dopant emission centre. The inverse relationship between the number of charge carriers generated in the cascade and the material band gap (eqn (2)) causes this wide gap host to limit the maximum number of generated emissive states. Secondly, carriers must diffuse to reach activation sites, increasing the time of scintillation and the probability of reaching a trap state. This leads to slow responses (for example 1 μs in CsI:Tl and 3 μs in GOS:Pr)^47^ and afterglow, while competing carrier trapping events can also quench carriers and reduce light yields. The efficiency of the energy migration phase, combined with the subsequent energy transfer to the activator site, is represented by the *S* term in eqn (1).

Intrinsic emission from direct band gap semiconductors, including PVKs and other emerging materials such as HgI_2_, PbI_2_ and II–VI semiconductors, occurs from band-to-band or excitonic states (Fig. 2b). Intrinsic emission allows narrower band gaps, increasing the maximum light yields. The absence of a transfer step in PVKs makes scintillation time scales limited by the emission step, and parity-allowed band-to-band or excitonic decay times can be less than nanoseconds. This makes PVKs potentially brighter and faster than traditional extrinsic materials; MAPbI_3_ materials can generate a theoretical maximum of ∼270 000 charge carriers per MeV, over 4 times as many as CsI:Tl. Combined with the high atomic weights of key components, including lead (*Z* = 82), caesium (*Z* = 55) and iodine (*Z* = 53), this makes PVKs well suited to the application of indirect radiation detection.

### 2.2 Performance requirements

The broad uses of ionising radiation give an equally broad range of requirements for scintillators (see Table 1). An important parameter is the incident radiation energy, which dictates the scintillator thickness and influences the subsequent figures of merit displayed by a device (see Table S1, ESI†). It is also important to consider the indirect detection system as a combination of the scintillator with the photodetector and signal processing electronics. The emission from the scintillator must be detected by the photodetector, which requires efficient spectral overlap of the RL and the photodetector quantum efficiency, and appropriate optical coupling between the two devices.

**Table 1:** Applications of ionising radiation detectors and the performance requirements

Application	Energy range (keV)	Typical material	Current performance			Application specific requirements
			Light yield (photons per MeV)	Decay time (ns) (afterglow)	Emission maximum (nm)	
Radiography (Medical)	60–120	CsI:Tl	60 000 [55]	1000 (0.5% at 3 ms) [55]	550	• Spatial resolution
						• Linear response with dose
						
Radiography (Industrial screening (food))	10–120	GOS:Tb	60 000 [56]	600 000 (<0.1% at 3 ms) [57]	545	• Scalable to large dimensions to image large objects
						• Emission wavelength suitable to silicon imagers
						• Low cost
						
Computed tomography (Medical)	80–140	CdWO_4_	20 000 [58]	2000 (0.05% at 3 ms) [59]	495	• Temperature coefficient < 0.1% °C^−1^ from beam induced heating
						• Afterglow < 0.1% at 3 ms
						• Decay times below 10 μs to excced sampling rates
						• Light yields > 20 000 photons MeV^−1^ for good signal to noise
						
Mammography (Medical)	20–40	a-Se (direct detection)	n/a	n/a	n/a	• Spatial resolution ∼100 s μm (to detect low contrast tumours and microcalcifications)
						
Positron emission tomography (Medical)	511	LSO:Ce	40 000 [60]	40 [60]	420	• Spatial resolution to accurately
						• Determine line of response
						• Energy resolution to reject scattered events
						• Short emission decay times to reduce coincidence gate
						
TOF-positron emission tomography (Medical)	511	LaBr_3_:Ce	61 000 [61]	35 [61]	358	• Timing resolution of 10 ps
						
Astronomy	3–79 (NuSTAR)	CdZnTe (direct detection)	n/a	n/a	n/a	• Energy resolution of ∼1.5% at 60 keV
						
Calorimetry (High-energy physics)	Photons and electrons	Many: PbWO_4_ (LHC calorimetry)	140 [62]	Several components: <10 ns, 20–200 ns, >500 ns [62]	475	• Energy resolution
						• Large area fabrication 2 × 2 × 20 cm
						• Sensitive to high energy photons
						
Gamma spectroscopy (Nuclear security)	10–10 000	NaI:Tl	43 000 [63]	230 [64]	415	• Energy resolution to resolve isotopes <7% 662 keV
						• Low cost for large areas

Two essential attributes of a scintillator are bright and fast emission. The emission from a scintillator is quantified by its light yield, *i.e.*, the number of photons emitted per MeV of incident radiation. Widespread commercial materials CsI:Tl and GOS:Tb both have light yields exceeding 60 000 photons MeV^−1^,^48^ allowing sensitive detection. Fast emission is a requirement in many applications, particularly imaging with a high frame rate (CT) and to achieve high timing resolution (TOF-PET). Currently, fast emission is offered by the 5d–4f transition in lanthanides (Ce^3+^, Pr^3+^, Eu^2+^),^49,50^ which offer decay times on the nanosecond scale whilst maintaining high emission yields, for example LSO:Ce (Lu_2_SiO_5_), LYSO:Ce and LaBr_3_:Ce (Table 2). While the exact requirements differ between applications, scintillators must also be radiation-hard to withstand high-energy photon exposure, and be able to tolerate any environmental and mechanical stress that is required under operation. The high energy of the incident radiation is known to damage existing scintillators, generating defects which promote non-radiative losses and increase afterglow, as well as colour-centres that increase parasitic absorption.^41^ CsI:Tl based flat panel imagers are expected to lose sensitivity with repeated radiation exposure, and are warrantied up to 8730 Gy with <100 kV X-rays.^51^ Many existing scintillators are also hygroscopic (for example CsI:Tl/Na, NaI:Tl and LaBr_3_:Ce) which requires additional packaging and storage considerations.

**Table 2:** RL decay times of fast commercial materials and PVK scintillators

Material	Decay time (ns)	Ref.
CsI:Tl	1000	55
LSO:Ce	40	60
LYSO:Ce	33	83
LaBr_3_-Ce	15	61
LuI_3_-Ce	31/140/1000	84
LuAP:Ce	17	85
BGO	300	86
BaF_2_^a^	0.8/630	87
CsPbBr_3_ NCs	44.6	38
CsPbBr_3_ (7 K)	1 (Fast component)^b^	82
MAPbBr_3_ (77 K)	0.1/1	37
PhePbBr_4_	9.4	66, 80 and 81
	9.9	
	11 (81%)/36 (18%)/236 (1%)	
CsPbBr_3_:Cs_4_PbBr_6_	3^c^	73 and 74
	1.4 (88%)/6.7 (12%)	

a Low light yields from fast crossover component and slow excitonic emission limit commercial use.

b μs background emission component also seen below 70 K that would contribute to afterglow.

c Measured with UV excitation. It has been reported that the decay time measured from RL excitation is slower, due to the additional excitation and cascade steps.^79^

The use of ionising radiation to generate images is a key application of detectors, requiring high spatial resolution, linear response with incident intensity and low levels of afterglow. In general, the spatial resolution of indirect detectors has a lower fundamental limit than direct detectors due to light scattering in the scintillation step. To mitigate these losses, CsI:Tl can be evaporated into large needle like structures that aid waveguiding and improve spatial resolution. Such detectors typically use a Fibre Optic Plate (FOP) as a supporting structure, which has the added advantage of attenuating the primary X-ray beam, providing protection for the optical sensor. In addition, high light yields (∼60 000 photons MeV^−1^) and mature fabrication techniques make CsI:Tl common in flat-panel imagers for medical radiography. Linear responses to incident intensity allow intensity discrimination between slight tissue density differences and such responses are primarily implemented by computed tomography, which has the benefit of consolidating large image sets, thereby reducing noise. Finally, a key requirement for imaging is low afterglow levels, which can distort image quality and reduce the modulation transfer function (MTF), especially in high frame-rate applications. Afterglow in CsI:Tl is high,^52^ limiting its use to longer integration times, with >2% emission remaining at 3 ms.^53^ Gamma-ray spectroscopy, security applications and high-energy physics all require the resolution of individual photon energies. Generating spectroscopic information requires operation of the detector in photon counting mode. The response of the scintillator must be proportional to the incident photon energy, and each photon must be analysed before the next one arrives, necessitating scintillator detectors with fast emission and fast processing electronics. The resolving ability of a scintillator is quantified by its energy resolution and an ideal indirect detector can resolve closely spaced gamma photons, such as the 604 and 662 keV emission of Cs isotopes.^46^ This requirement is particularly important in security applications and is fulfilled by NaI:Tl, with energy resolution around 7%^54^ (see ESI† for Definition) as well as a large light output of 43 000 photons per MeV, and the ability to be fabricated in large dimensions.

## 3. PVK scintillator performance

### 3.1 Stopping power

**Fig. 3:**
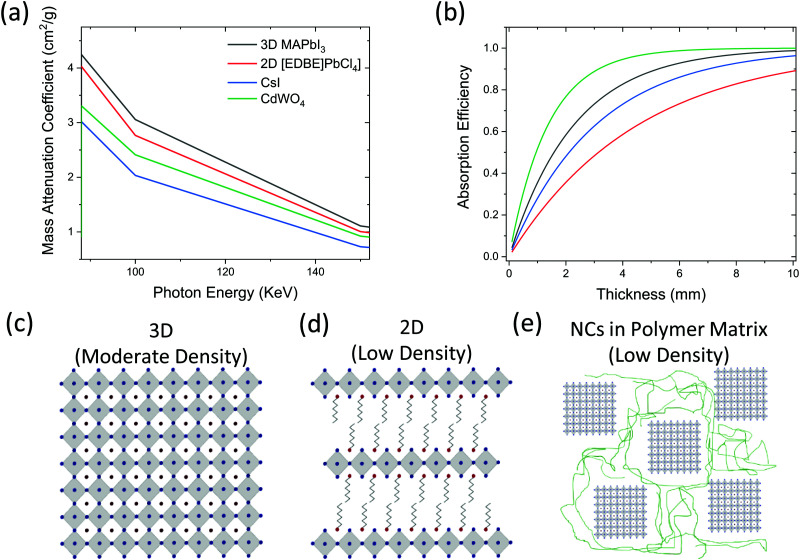
(a) Mass attenuation coefficient variation with photon energies relevant to medical imaging for PVK scintillators and commercial CsI and BGO materials (calculated in ref. 65) and (b) the corresponding absorption efficiency once material density is accounted for at 150 keV. The low efficiency of 2D PVKs to attenuate the incident photons highlights the requirement of high density materials (note legend is common in (a) and (b)). (c–e) Crystal Structure schematics demonstrating the detrimental effect of reducing dimensionality on material density by introducing low *z* organic components as a-site cations (d) or embedding nanocrystals in a polymer matrix (e).

PVKs have high mass attenuation coefficients due to their high-*Z* components. As a result, at 100 keV, a relevant energy for many imaging applications, the coefficient for MAPbI_3_ is 3.1 cm^2^ g^−1^, in comparison to 2.0 cm^2^ g^−1^ for CsI:Tl (Fig. 3a). However, the stopping power of a scintillator depends on the mass density of the material, not just the mass attenuation coefficient. The attenuation of photons through a solid can be obtained using the Beer–Lambert law (eqn (3)) which shows the dependence of the intensity of transmitted photons *I* on the material density *ρ*3
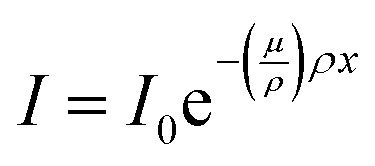
where *I*_0_ is the intensity of incident photons, *μ* is the linear absorption coefficient, and *x* is the material thickness. The mass attenuation coefficient, *μ*/*ρ*, is often used due to its lack of material density dependence, allowing comparisons regardless of phase or crystal structure.^41^ However, the material mass densities of PbWO_4_ and BGO (Bi_4_Ge_3_O_12_) exceed 7 g cm^−3^, compared to generally less than 4 g cm^−3^ for PVK single crystals. This is in part due to the large volume occupied by the halide ions; in comparison, oxide perovskites scintillators reach higher values (LuAlO_3_ – 8.3 g cm^−3^) (Fig. 3c). Although densities around the value of PVK single crystals are observed in traditional scintillators (NaI and CsI crystals are 3.7 and 4.5 g cm^−3^, respectively), some of the most promising PVK materials are even less dense. For example, the highest light yields to date have been demonstrated with PVKs of reduced dimensionality. 2D materials contain alternating inorganic layers with large organic spacers, and these organic moieties reduce the density further compared to 3D analogues (Fig. 3d), with (NH_3_(CH_2_)_2_O(CH_2_)_2_O(CH_2_)_2_NH_3_)PbCl_4_ ((EDBE)PbCl_4_) yielding just 2.2 g cm^−3^.^52^ Similarly, despite bright RL demonstrated from PVK nanocrystals such as CsPbBr_3_, polymer host matrices for the nanocrystals are often used, reducing density further (Fig. 3e). The low-*Z* organic components of the matrices increase the attenuation length, and any improvements in film quality or environmental stability provided by the polymer must be carefully balanced against the increase in penetration depth in the scintillator. The decrease in stopping power in reduced dimensionality systems can be seen in Fig. 3b, where almost twice as much material is needed with a 2D perovskite compared to its 3D analogue to attenuate the 150 keV photons utilised in medical CT scanners. This reduces the image resolution of the detector due to an increase in light scattering inside the scintillator generating optical crosstalk.

### 3.2 Light yields

**Fig. 4:**
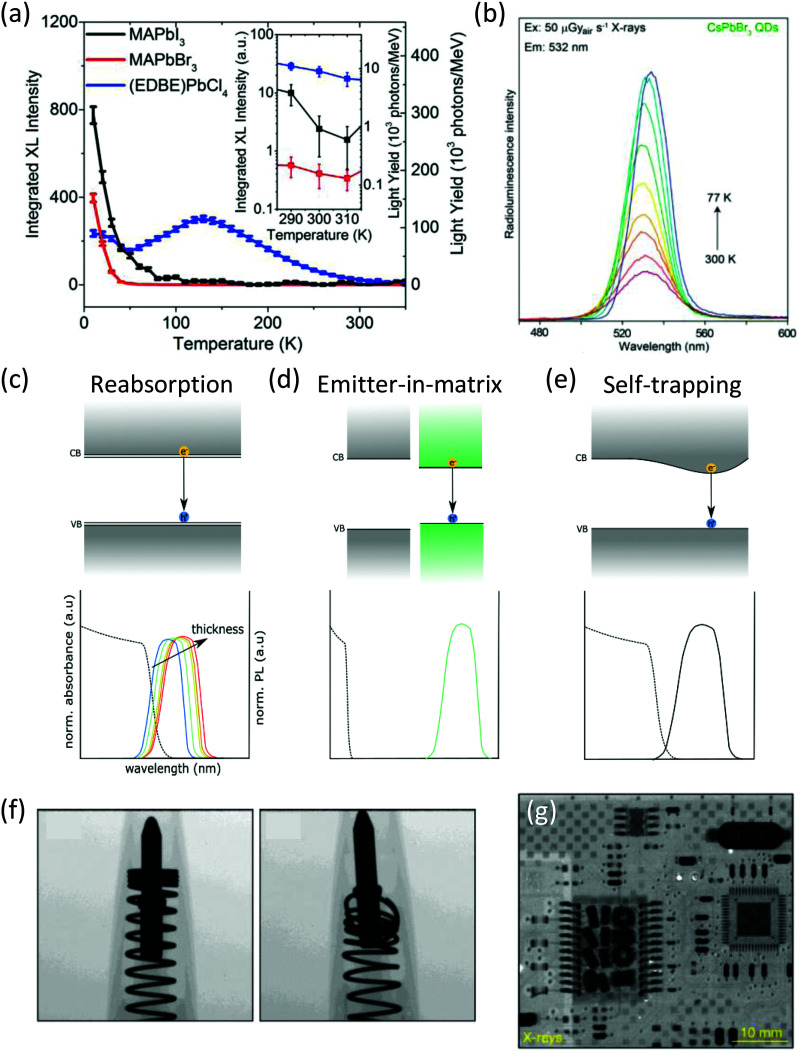
(a) Temperature and dimensionality dependence of PVK RL from 3D MAPbX_3_ single crystals, and 2D (EDBE)PbCl_4_, showing higher room temperature emission from the reduced dimensionality system. Reproduced from ref. 52 with permission from Springer Nature. (b) Temperature dependence of CsPbBr_3_ NC RL. Reprinted with permission from Springer Nature.^38^ (c–e) Schematics of energy levels and expected absorption (dashed lines) and emission spectra (solid lines), highlighting the self-absorption issue with PVKs and possible solutions. (c) Band-to-band emission with significant overlap between the absorption and emission spectra. The normalised emission as thickness increases shows the expected redshift due to reabsorption of the shorter wavelengths. (d) Reintroducing the extrinsic mechanism with an emitter-in-matrix system. (e) Self-trapped exciton emission with large Stokes shift. (f–g) Images taken with PVK scintillators (f) CsPbBr_3_ NCs imaging a ball point pen. Reproduced from ref. 39 with permission from Wiley. (g) CsPbBr_3_ NCs imaging a network interface card. Reprinted with permission from Springer Nature.^38^

Reports of room temperature light yields in PVKs have thus far been limited to reduced dimensionality systems, either through compositional or physical confinement, and this is proposed to be due to the increase in exciton binding energy preventing thermal quenching of the emissive states at room temperature (Fig. 4a). 2D PVKs (C_6_H_5_(CH_2_)_2_NH_3_)_2_PbBr_4_ ((Phe)PbBr_4_) and (EDBE)PbCl_4_ have displayed light yields of 10 000^66^ and 9000^52^ photons MeV^−1^, respectively. Similarly, all-inorganic CsPbBr_3_ systems have displayed RL, with nanocrystals (NCs) showing 5× higher emission intensity than YAlO_3_:Ce systems,^38^ and nanosheets^67^ yielding 21 000 photons MeV^−1^. In contrast, bulk 3D systems such as MAPbBr_3_ single crystals, whose growth into large area thick layers is already well documented,^68^ have yet to display any appreciable scintillation at room temperature,^36^ with bulk CsPbBr_3_ emission intensities around 4 orders of magnitudes lower than CsPbBr_3_ NCs under the same conditions.^38^ The greater exciton binding energy of the NCs (up to 120 meV) compared to bulk PVK systems (<15 meV^69^) was reasoned to prevent thermal quenching of the emissive state. A strong temperature dependence of light yield has been found in PVK materials^70^ (Fig. 4b), allowing bright emission even from 3D systems at low temperatures.^52^ Light yields of 90 000 ± 18 000 and 116 000 ± 23 000 photons MeV^−1^ at 77 K and 8 K respectively have been demonstrated in MAPbBr_3_ single crystals,^37^ consistent with a freezing out of the thermal dissociation of excitons, which when combined with the low trap densities (∼10^10^ cm^−3^)^71^ promotes efficient radiative recombination.

While promising, the room temperature light yields of PVKs are suggestive of losses, and parasitic self-absorption of the emitted photons will limit external emission of the photons (*i.e.*, RL intensities). For example, the intrinsic emission spectrum of direct band gap semiconductors with small Stokes shifts, such as 3D PVKs, overlaps with their absorption spectra, and this causes significant reabsorption and thus limits light yields (Fig. 4c). Self-absorption in thick PVK materials was originally studied under optical excitation, where Wenger *et al.*^72^ found that increasing thickness decreases the intensity and redshifts the photoluminescence emission through reabsorption particularly of the higher energy photons. The external photoluminescence quantum efficiency (PLQE) of 2 mm-thick MAPbBr_3_ crystals was reported to be 6.4% despite internal values calculated to be 67%, and reducing this disparity has since become a focus in the scintillator community.

Solutions to reduce the impact of reabsorption have exploited the embedding of the PVK emitter within a wide band gap matrix (Fig. 4d). This includes CsPbBr_3_ NCs contained in a wide band gap Cs_4_PbBr_6_ matrix, where the matrix contributes to the stopping power and helps stabilise the emission centres.^73^ The strong confinement in the 0D structure yields an exciton binding energy of ∼90 meV and, combined with reduced self-absorption, led to light yields of 64 000 photons MeV^−1^ at room temperature.^74^ Similarly, an organic dye with a large Stokes shift has been combined with CsPbBr_3_ NCs, where efficient sensitisation by the PVK and emission by the organic component at longer wavelengths^75^ minimises reabsorption losses. However, the final device offered only moderate RL intensity, comparable to BGO (8200 photons MeV^−1^). A similar system was proposed by Ning *et al.*^76^ for visible light conversion, where PbS quantum dots were fabricated within a perovskite, with the NIR emission from the quantum dots shifted well away from the PVK absorption onset, minimising reabsorption. Localisation and relaxation of emissive species with the lattice can induce a Stokes shift, and negligible self-absorption was demonstrated in 1D structured Rb_2_CuBr_3_ due to self-trapping of excitons^77^ (Fig. 4e). The exceptional PLQE values of 98.6% translated to approximate light yields of 91 000 photons MeV^−1^, the highest room temperature value from PVK scintillators to date. Emission from self-trapped excitons was also seen in the Cs_2_Ag_0.6_Na_0.4_In_1−*y*_Bi_*y*_Cl_6_ double perovskite,^78^ where the Bi^3+^ doping can be used to tune the radiation stopping power due to its high atomic number. An optimum of 15% loading produced light yields of ∼39 000 photons per meV. However, these self-trapped excitonic systems are prone to longer decay times on the order of tens of microseconds, with considerable afterglow in Rb_2_CuBr_3_ of 2.72% at 20 ms, and thus may be more suitable for a more limited range of applications.

### 3.3 Response speeds

PVK scintillation has been shown to occur very rapidly, with fast decay times presented in literature. CsPbBr_3_ nanocrystal scintillators demonstrated a short decay time of 44.6 ns under gamma excitation.^38^ The same material displayed a 5 ns decay time upon UV excitation, with the faster decay potentially due to avoiding the additional excitation and cascade steps in RL,^79^ combined with the shorter penetration depth of UV compared to X-rays exciting the surface. The decay time shortened to 3 ns once combined in a Cs_4_PbBr_6_ matrix, attributed to additional confinement accelerating recombination.^73^ A similar CsPbBr_3_:Cs_4_PbBr_6_ system has recently offered impressively short RL decays, with a 1.4/6.7 ns fast and slow component, showing promise for fast detection applications.^74^ 2D perovskites have displayed rapid room temperature decay times, with (EDBE)PbCl_4_ demonstrating a single decay component of 7.9 ns from UV-Vis excitation,^52^ and (Phe)PbBr_4_ has been measured independently to have a dominant decay time of around 10 ns,^80,81^ offering considerable improvement on current materials (Table 2). Studying the scintillation temperature dependence from MAPbBr_3_ single crystals, Mykhaylyk *et al.*^37^ demonstrated that above 60 K the emission components retain impressive decay times of 0.1/1 ns respectively, which combined with increased light yields of ∼90 000 photons MeV^−1^ at liquid nitrogen temperature make this a prospect for ultrafast timing precision. Similarly, RL from CsPbBr_3_ single crystals reached 1 ns at 7 K, and outperformed LYSO:Ce at resolving a pulsed synchrotron beam.^82^ Detector cooling is already a feature in HP–Ge direct detectors, operated at 77 K due to the small material band gap, and so is a commercial possibility for PVKs.

### 3.4 Stability

The tolerance of PVK scintillators to X- and gamma-rays has not been studied in detail, though initial results look promising. Reports have demonstrated steady RL intensity under continuous irradiation for several hours in CsPbBr_3_ NCs^38^ and nanosheets (2 hours at 18 μGy s^−1^).^88^ Higher doses have also been tolerated by PVK scintillators, with Cs_2_Ag_0.6_Na_0.4_In_0.85_Bi_0.15_Cl_6_ showing negligible losses after 50 hours and a 34 Gy cumulative dose,^78^ whereas 800 Gy reduced the RL intensity of CsPbBr_3_ NCs with an organic dye to just 85% of its initial value.^75^ Although reductions in light output begin to show at these doses^74^ – tridoped Cs_2_Ag_0.6_Na_0.4_InCl_6_:Yb^3+^/Er^3+^/Bi^3+^ reduced its RL to 49% after 53 Gy^89^ – they still outperform traditional materials, as CsI:Tl emission dropped to 28% after the same dose. An improved understanding of the degradation mechanisms from more focussed studies correlating the effect of the high-energy radiation on both the PVK material and the scintillation performance would benefit the field enormously. The stability of PVKs in direct detectors may also provide useful understanding in this context. A perovskite-in-polymer membrane system showed negligible current reduction after a total dose of 376.8 Gy (equivalent to 1.88 million chest X-rays)^90^ while MAPbBr_3_ single crystals irradiated with a 230 Gy dose over 10 hours displayed no losses.^91^ The retention of charge transport properties would suggest no significant material degradation, which is promising for scintillators.

There are also studies on the effect of high-energy radiation on PVK solar cells. The importance of efficiency, weight and size have made PVKs a promising prospect for space applications, and as such there is are efforts to analyse the tolerance of these materials to radiation. Gamma-ray studies have shown small losses in *J*_sc_ in mixed-halide systems, due to radiation-induced phase segregation,^92^ similar to the Hoke effect,^93^ although the largest losses are due to colour centers in the glass reducing transmittance.^94^ The broad quantum efficiency of most photodetectors used in combination with scintillators makes precise tuning of the band gap by alloying different halides less important than in solar cells; as a result this phase segregation should not impact PVK indirect detectors. A range of PVK absorbers were tested by Boldyreva *et al.*,^95^ with MAPbI_3_ displaying remarkable stability after 1 MRad (equivalent to 10 kGy) of gamma exposure, attributed to CH_3_I and NH_3_ undergoing radiation-induced radical chemistry that then self-heal any generated defects. Similarly, MAPbI_3_ was tested in an energy converter application, and devices showed no performance degradation after a 57 Gy gamma dose (57 Sv H*(10)).^96^ The radiation hardness of PVKs has also been shown to extend to protons and electrons, demonstrating a similar self-healing behaviour after irradiation.^97,98^ It is proposed that the lability of the constituent ions in PVK structures allows any defects formed by the radiation to be removed, limiting and recovering from, any damage. In addition, many defects in PVKs introduce only shallow states, and so radiation-induced defects may be benign to operation.^99^ The moisture instability of PVKs may require encapsulation in end designs, which may further complicate outcoupling considerations. However, many current materials suffer from hygroscopicity^100^ and detector designs often guard against this.

### 3.5 Full device performance

Despite a short period of development, PVK scintillators have already been incorporated in full indirect detection systems, displaying impressive performance. High MTF values have been reported in NC-based PVK imagers, which exhibit reduced internal light scattering compared to bulk films and single crystals. The resulting reduction in optical crosstalk is promising for high-resolution imaging applications. MTF values of 9.81 lpmm^−1^ (MTF = 0.2)^39^ and MTF = 0.72 at 2 lpmm^−1^ ^38^ have been reported in CsPbBr_3_ NCs, exceeding the performance of GOS and CsI:Tl reference devices, respectively. Examples of images produced with PVK scintillators are shown in Fig. 4f–g. The short decay times of the Cs_2_Ag_0.6_Na_0.4_In_1−*y*_Bi_*y*_Cl_6_ double perovskite enabled a dynamic image of a bending finger to be produced, possible at low incident doses of 47.2 μGys^−1^.^78^ However, initial resolution of 4.3 lpmm^−1^ (MTF = 0.2) dropped to 1.4 lpmm^−1^ as wafer thickness increased from 0.1 to 0.6 mm due to increased light scattering within the scintillator. Linear responses between 30 – 550 μGys^−1^, the limits of the X-ray source utilised in the study,^77^ have been presented, as well as linearity down to 13 nGys^−1^, an encouraging result for low dose imaging.^38^ Apart from self-trapped PVK emission, reports on PVKs have displayed virtually no afterglow (5 ppm at 20 ms^81^), offering levels similar to commercial BGO and CdWO_4_. Combined with the short decay times, this may open up use in even faster frame rates than is currently possible.

There have also been reports of energy resolution from PVK spectroscopic systems. For example, (Phe)PbBr_4_ crystals exhibited an energy resolution of 35 ± 5% (662 keV) with a linear response over the 122–662 keV range,^81^ and a similar lithium doped (PEA)_2_PbBr_4_ (PEA = phenethylammonium) system presented 12.4% (662 keV).^101^ In addition, MAPbBr_0.05_Cl_2.95_ demonstrated an improved 10.5 ± 0.4% (662 keV).^102^ A CsPbBr_3_:Cs_4_PbBr_6_ powder presented the best energy resolution values in PVKs to date of 3.0 ± 0.1% (59.6 keV) which shows promise in this application.^74^ Overall, although current performance falls short of specific energy resolving detectors, such as Silicon Drift Detectors (SDDs), the energy resolution of PVKs is largely unexplored and represents an exciting area for further development.

## 4. Outlook

PVKs have thus far shown enormous promise as scintillating materials, demonstrating good light yields and impressively short decay times, all from low temperature solution synthesis. However, there remains plenty of room for improvement in their performance metrics to reach their potential, while selecting the applications where PVKs can have the most impact requires consideration.

### 4.1 Improving scintillator light yields: learning lessons from PVK photovoltaics and LEDs

The large cascade of charge carrier generation gives PVK scintillators high theoretical light yields, but to date recorded values are much lower. Common themes emerge to explain these losses: competing non-radiative recombination processes lowering internal light yields, and outcoupling issues, due to reabsorption preventing efficient light escape. Solutions to these challenges could be found within the field of PVKs for solar cells and LEDs where maximising radiative efficiency and optoelectronic performance is paramount.

#### 4.1.1 Internal light yields

The external photoluminescence quantum efficiency (PLQE), the fraction of photons emitted by a sample relative to the number of photons absorbed, is a common metric used to assess the radiative efficiency of optoelectronic devices. A key difference between RL and PL is the direct excitation of the excited state in PL measurements with photons of energy comparable to the band gap, whereas RL includes the initial interaction of the high-energy ionizing radiation followed by the resulting cascade and relaxation steps. As a result, losses in this initial scintillation step are not the focus of PL improvement. The spectra of PVK emission after visible and X- and gamma-ray excitation are very similar,^103^ albeit typically with a redshift of the RL due to the self-absorption of the higher energy emission that is well documented in thick PVK samples, suggesting that both PL and RL are likely to originate from the same states. However, the recombination regime and thus carrier lifetime are both strongly dependent on the charge density and therefore the relevant luminescence efficiency metric will depend on the excitation conditions relevant to the application.^104^ For example, the PLQE of photovoltaic materials is typically assessed under “1-sun” illumination conditions, which generates an initial charge-carrier density of ∼10^15^ to 10^16^ cm^−3^ and trap-assisted recombination is the dominant non-radiative process that competes with the radiative bimolecular component.^104^ As a result, research in the solar cell field has focused on reducing the density of trap states *via* passivation to improve luminescence yields. However, in operating LED devices, charge densities are typically higher and non-radiative Auger recombination also needs consideration. In contrast, the lack of standard excitation conditions for radiation detectors, with the incident beam energy and flux depending on the application, combined with the complicated cascade of charge carrier generation for every incident photon, makes it difficult to calculate an expected carrier density regime in scintillators. Auger recombination processes will lead to non-proportional effects in energy resolved detectors at high incident energies.^57^ In such cases, high mobility materials have been proposed to rapidly diffuse carriers away from high density regions and ensure radiative recombination competes sufficiently with non-radiative Auger processes.^105^ In addition, it is likely that the large material volume and high trap densities in PVK scintillators will still allow trap-assisted recombination to contribute to emission losses in many applications. Therefore, applying techniques established to improve PLQE may have benefits in increasing the emission quantum efficiency of the final step in RL, and improve PVK scintillator light yields.

Removing trap states *via* passivation is common in PVK thin films to overcome the high defect densities,^106,107^ with a wide range of methods shown to raise emission efficiencies, demonstrating internal PLQEs exceeding 95%.^108^ Successful approaches include adding passivating agents to the precursor solution,^108,109^ chemical post treatment^110,111^ and light soaking,^112,113^ with species such as phosphate based molecules binding to uncoordinated lead sites,^114^ 2D molecules forming well passivated quasi-2D perovskite surfaces,^115^ or alkali halides binding excess halide species.^108^ In thin-film devices, the short penetration depth of UV-Vis light and the importance of any interfaces with charge transport layers makes surface defects highly important, and often the target of passivation techniques. However, the increased thickness and deeper charge carrier generation in scintillators makes bulk trap states more relevant and passivation techniques targeting these bulk traps would be important. Systematic passivation approaches to PVK scintillators are yet to be reported.

Raising PLQEs by increasing the charge carrier density into the bimolecular regime is another prevalent tactic in solar cells and LEDs. Reducing the material volume in LEDs by decreasing PVK thickness can allow charge carrier densities to exceed trap-assisted recombination levels,^116^ but the necessity of thick materials to be sufficiently attenuating makes this approach impractical for scintillators. An alternative method to increase carrier densities involves the use of charge carrier funnelling to enhance radiative efficiency. Quasi-2D materials, with *n* (*n* > 1) layers of PbI_6_ octahedra between large organic spacers contain a mixture of *n* layered phases, with carrier funnelling to the lowest band gap occurring. PLQEs above 10% at low excitation fluences of 6 mW cm^−2^ resulted from the high carrier concentrations (∼10^16^ cm^−3^) within these low band gap phases, translating into efficient LED devices.^117^ The lower energy emission from the smallest band gap would also contribute to reduced reabsorption. However, the compositional engineering of these phases is complex, and can introduce additional defects and grain boundaries that limit radiative efficiency, and require additional passivation.^114^ This may add complications when scaling up to the large volume of material needed in radiation detection. A solution may come from the finding that mixed halide PVKs can self-organise into band gap gradients, an effect that can be stabilised by cation selection, to achieve similar charge carrier concentration but with greater potential for upscaling;^118^ more work is needed to control such gradients and implement into scintillators.

#### 4.1.2 Light management and outcoupling

Maximising the extraction of light from the scintillator is a key area of research, largely due to the parasitic self-absorption of emitted light by PVKs. Solutions are being developed (*cf.* Section 3.2), such as introducing a transfer step to a redshifted emission centre or engineering materials with large Stokes shifts by the self-trapping of emissive moieties. Reintroducing an extrinsic emitter system nullifies the inherent advantages of PVK scintillators. Firstly, wide band gap hosts require an additional charge carrier transfer, and secondly the light emission from the scintillator is restricted to longer wavelengths, limiting the compatibility with standard visible photodetectors. Therefore, designing PVK scintillators with sufficient Stokes shifts should provide the more fruitful approach to retain the inherent advantages of PVKs. However, the contribution of photon recycling, and the possibility of photons escaping after multiple emission and reabsorption events, has not yet been considered in PVK scintillators. Photon recycling has been demonstrated in PVK solar cells, allowing high excitation densities to increase quasi-Fermi-level-splitting, and subsequently produce high V_oc_ values.^119^ Photon recycling has also been calculated to contribute >70% of light emission in state-of-the-art perovskite LEDs,^120^ and is partly responsible for their outstanding EQEs, by giving photons approaching the surface of the PVK outside the cone of emission another chance of escape. The extreme thicknesses required in scintillating materials compared to photovoltaic and LED systems make photon recycling considerations all the more relevant. It has been shown that the number of photon recycling events increases with thickness in a solar cell operated at maximum power point, and this would increase further in a scintillator system due to the absence of charge carrier collection.^121^ However, while many recycling events may occur in scintillators, extracting sufficient numbers of photons in the forward direction from samples of relevant thicknesses may still prove difficult without shifting the emission energies away from the absorption edge of the majority of the material. Even with a straight-line propagation of reemitted light, the optical extinction length of the scintillated light would require around 385 photon recycling events to travel across a 270 μm CsPbBr_3_:Cs_4_PbBr_6_ film.^122^ Internal PLQEs of 95% would still result in the quenching of almost all emission, even with this conservative estimation of the number of recycling events. As a result, to achieve high light yields in PVK scintillators, preventing reabsorption is an essential design feature and this must be balanced with the desired properties of the detector systems, *e.g.* retaining fast emission.

While photon recycling allows additional attempts at emission, better outcoupling increases the probability of emitted photons escaping the scintillator and reaching the photodetector. This is achievable using photonic structures, which have recently emerged as a concept to improve the performance of existing high refractive index scintillators.^123–126^ Increasing the cone of emission will improve light yields of scintillators, with simulations suggesting 90–110% enhancements,^124^ and increase the timing resolution, by increasing the likelihood that light is emitted at the first instance it meets the scintillator/detector interface, increasing the light yield at early times.^123^ Spatial resolution would also improve through decreased lateral distances between the point of emission and detection by the photodetector. Better optical coupling between the scintillator and detector can provide similar performance improvements, possible in PVK devices by direct deposition onto photodetectors (discussed in Section 4.2.2) or on to fibre-optic plate structures. These performance benefits are of relevance to PVK scintillators yet such approaches remain unexplored. There have been examples of photonic structures improving the performance of PVK lasers^127,128^ and also coloured solar cells.^129^ Extending this to scintillators would be a fruitful prospect, and could prove simpler due to the absence of any additional transport layers on the surface.

### 4.2 Applications and opportunities

Besides the exceptional emission properties, PVK materials also have unique features, such as band gap tunability and solution processibility, which provide further advantages over existing materials and potential opportunities for novel detector applications.

#### 4.2.1 Tunable emission

By varying the constituent ions and the material dimensionality during synthesis, the properties of PVKs can be tuned. This allows the band gap to be controlled, and consequently the emission wavelength can be selected to suit the required photodetector for applications. Important photodetectors are photomultiplier tubes (PMTs) and silicon photomultipliers (SiPMs) for high sensitivity and photon counting modes, and silicon detectors in imaging arrays (CCDs/CMOS). CsPbX_3_ NCs, as one example, can be optimised to each of these applications by varying the halide ion, with CsPbCl_3_ optimised to ∼400 nm for bi-alkali photocathode PMTs, while CsPbBr_3_ would suit the peak sensitivity of silicon detectors (∼550 nm). The large degree of band gap tuning offered by PVKs has other commercial applications, by adding spectral information onto images with multispectral scintillators. This would be applicable to energy-selective CT scanning; where traditional CT displays a contrast image based purely on the different attenuation of materials, spectral CT collects the energy dependence of the attenuation by splitting the detector into two scintillators, a top layer optimised to soft radiation and a bottom layer for hard radiation.^130^ This provides information on the atomic number of the tissues in the scan, and is especially useful when radiocontrast agents such as iodine are added. It also opens up the possibility of applications which traditionally use DEXA (dual energy X-ray absorptivity), such as bone densitometry. Utilising the range of PVK scintillators in a multispectral system was reported by Sytnyk *et al.*^40^ who proposed a four layer stack of PVK materials, with each layer utilising a different PVK in order to provide a greater spectral resolution. Each layer is optimised in terms of stopping power to a range of incident X-ray energies, and each emits at a different wavelength. When combined with a spectrally resolved detector, or a a-Si:H array with colour filters, this could enhance current ionising radiation imaging.

#### 4.2.2 Low temperature fabrication and flexible detection

A desirable feature of PVKs compared to other scintillator materials is their simple fabrication, retaining exceptional optoelectronic properties when deposited from solution. Solution processing and low temperature material growth simplifies film fabrication, reduces production costs and enables compatibility with a range of substrates and electronics. It allows growth directly onto photodetectors or fibre optic plates, improving optical coupling with the emitting scintillator, with demonstrations on pixelated α-Si photodiode arrays for imaging^38^ and silicon photomultipliers (SiPM)^102^ without optical grease. Existing scintillators, such as NaI:Tl and BGO for spectroscopy, are grown in single crystal form at high temperatures (>1700 °C) with high production costs. CsI:Tl for imaging screens involves an expensive vacuum evaporation growth to obtain its needle-like morphology. GOS phosphors are a cheaper alternative, but at a reduced sensitivity and spatial resolution. PVK synthesis has shown to be easily controlled to produce the structures required for detector applications. PVK single crystals can be quickly grown using inverse temperature crystallisation, utilising the reduction in solubility with temperature displayed in some solvents,^131,132^ and the growth shown to be influenced by the shape of the container.^131^ This allows both large area and sub-micrometre crystals to be produced.^133–135^ Furthermore, the control of perovskite morphology *via* different processing routes could allow for improved high-resolution imaging, and a low cost alternative to CsI:Tl. Grain size engineering^136,137^ or optimisation of NC films to further reduce light scattering and optical crosstalk may enable further advances in MTF values.

**Fig. 5:**
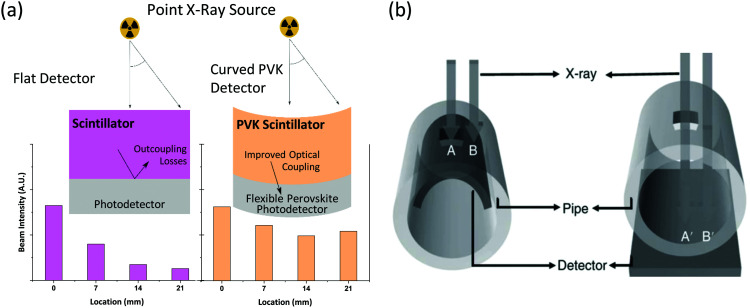
Potential for unique functionality with flexible PVK scintillators. (a) Overcoming uneven X-ray intensity distributions from point sources with flexible PVK scintillators and photodetectors. Scintillators can be combined with flexible PVK photodetectors, which can simplify fabrication of the full indirect detector system and potentially improve light outcoupling from scintillator to photodetector. Adapted by permission from Springer Nature.^90^ (b) Flexible detectors conforming to the inside of a pipe allow lower energy radiation and consequently thinner scintillators with enhanced spatial resolution. Reprinted with permission from Springer Nature.^90^

Low temperature deposition also enables compatibility with flexible substrates, resulting in a detector that can conform to surfaces, useful in medical diagnostics and non-destructive testing.^138^ The importance of flexible radiation detectors was well explained by Zhao *et al.*,^90^ whereby intensity differences between pixels in the centres and edges of the detector from point X-ray sources can result in imaging errors and misdiagnosis^139^ (Fig. 5a). Substrate flexibility also allows better non-destructive industrial imaging by being placed inside of objects and thus requiring softer beam energies and thinner scintillators (Fig. 5b). Flexible PVK devices are well developed,^140^ with flexible solar cells exceeding 19% power conversion efficiency,^141^ and flexible direct X-ray detectors demonstrated.^142–144^ Retaining flexibility with increasing PVK thickness is a challenge, and a PVK in a porous nylon membrane, developed for flexible direct X-ray detection, demonstrated 2/3 mm minimum bending radii in 130/240 μm thick devices, respectively. However, unlike direct detectors and solar cells, which require charge transport considerations and compatible transport layers and contacts,^145^ scintillators are much simpler, making the prospect of developing a flexible scintillator promising. Alternate uses of flexibility could include accurately measuring dose delivery with detectors conforming to body parts. Despite lead-based PVK scintillators being exempt from EU regulations on hazardous substances in electronic equipment (RoHS),^146^ applications requiring closer contact with the human body may require less toxic analogues. Alternative metals such as copper, europium, bismuth and tin have all been integrated into perovskite-like structures and demonstrated scintillation.^78,147–152^ Lead-free indirect detectors have demonstrated exceptional performance, and currently present the highest light yields of PVK materials to date (Rb_2_CuBr_3_ ∼ 91 000 photons per MeV,^147^ Cs_4_EuX_6_ ∼ 78 000 photos per MeV^151^). However, the decrease in stopping power when replacing lead with a lower atomic number element must be considered.

There are significant cost reductions in the material production of PVKs compared to commercial scintillator materials, as cheap and earth-abundant precursor materials, combined with low temperature (<150 °C) and ambient processing conditions, contribute to inexpensive production. An analysis was presented by Cao *et al.*^73^ in their growth of CsPbBr_3_:Cs_4_PbBr_6_, suggesting the cost of a crude ingot was similar to CsI:Tl, while considerable savings up to a factor of 2000% can be made over Ce^3+^ doped materials (LYSO, YAG, YAP used for spectroscopic applications) without the concerns on the supply and extraction of rare-earth materials.^153^ High-throughput production of PVKs have also been projected to bring costs down further in other areas, for example in photovoltaics,^154^ where scalable techniques, such as slot-die coating,^155^ doctor blading,^24^ inkjet printing^156^ and thermal evaporation^157^ have all been demonstrated. It was estimated that detectors for CT, PET, SPECT (single-photon emission computed tomography) and high energy physics require over 70 000 kg of scintillator material annually at today's usage alone,^158^ emphasising the importance on the scalability of production.

#### 4.2.3 All-perovskite indirect detectors

The considerable development of PVK production techniques demonstrates another significant feature unique to perovskites that will suit their commercial success: the wide range of other PVK optoelectronic devices vying for market penetration. The difficulties in advancing from material discovery to large scale production is often the largest hurdle for new scintillators, due to difficulty in large-area growth and understanding the role of defects in relation to performance.^159^ It is of great advantage that PVKs share these challenges between multiple prolific fields, which should ensure continued rapid transition from the academic lab to commercial success. Further, the simultaneous development of PVK photodetectors can be specifically exploited for all-perovskite indirect detectors.^160^ The field of PVK photodetectors is very active, with impressive detectivities and response times^161^ and high gain factors over 10^5^ ^162–164^ suiting high sensitivity requirements. Flexible PVK photodetector arrays have already been demonstrated,^165^ highlighting the potential for an all-perovskite system to offer functionality beyond that of current materials, specifically for PET detectors. From a commercial perspective, these devices would also simplify detector fabrication, and could overcome outcoupling problems with refractive index matching to reduce internally reflected RL. Self-powered photodetectors^166–168^ would enable greater portability, by avoiding the need for bulky power supplies, improving access to radiation detector systems. This is relevant in healthcare, where patients may not have access to hospitals, as well as industrial radiography and mobile security, in conjunction with small and portable radionuclide gamma sources.

#### 4.2.4 Additional PET functionality

**Fig. 6:**
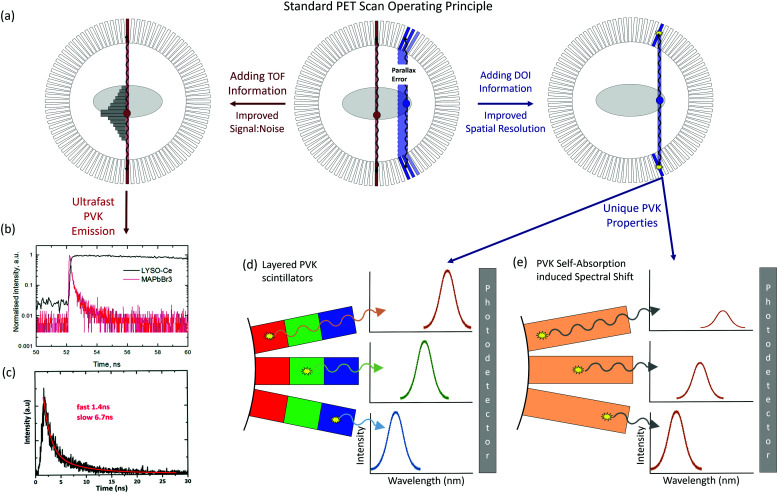
(a) The operating principle and existing issues of PET (centre) with parallax errors from off-centre detector line of response (LOR). Adding TOF information (left) provides information of the interaction position along the LOR, and depth information (right) increases spatial resolution. (b and c) Demonstrations of ultrafast PVK scintillation to provide TOF information. Reproduced from ref. 37 and 74, respectively, with permission from The Royal Society of Chemistry. (d and e) Utilising PVK to add depth information by (d) layering different band gap materials and (e) exploiting the spectral change from RL self-absorption.

An enormous performance advantage of PVK scintillators over current materials is their rapid emission times, improving detector timing resolution. Fast emission is required in positron emission tomography (PET), and exceeding the response times of the currently used scintillators can enable additional functionality with time-of-flight PET (TOF-PET). Traditional PET works using a radioactive tracer, designed to emit a pair of antiparallel 511 keV gamma-rays upon positron decay, to image and monitor biological pathways in the body (Fig. 6a). The pair of gamma photons is then coincidently detected by a ring of scintillator detectors. The position of the two photons will then produce a line of response (LOR) along which the annihilation occurred, and building up enough detection events produces an image. The desirable attributes of a PET detector are introduced In Table 1, and currently BGO, LSO and LYSO are commercially used in such systems. By adding additional timing information, the point of emission along the line of response can be known which can improve the signal-to-noise ratio of the system, especially in full body scans.^169^ The important parameter in TOF detectors is the timing resolution of the whole detector system, and for scintillator devices this is strongly dependent on the decay time and the light yield of the emission.^170^ A timing resolution of around 10 ps FWHM is the target, as this will allow a 1.5 mm confidence window of the interaction along the line of response.^57^ To achieve this, scintillator decay times below 1 ns, and a light yield of 10 000 photons per ns, are required.^171^ This has already shown to be possible in cooled perovskite single crystals,^37^ which may enable deployment in this role (Fig. 6b and c). Additional work towards fast, room temperature emitters will be further enabling.

PVK based detectors can also improve the spatial resolution of PET scanners by adding depth of interaction (DOI) information. The penetration of 511 keV photons requires long scintillator crystals which limits the spatial resolution, and causes parallax errors from off-centre photons not approaching the scintillator perpendicularly.^172^ This is particularly problematic in small ring systems. Adding depth information is possible with the wide range of PVK scintillator materials, with layers of two or more detector materials with different decay times or emission wavelengths, which can be distinguished by the photodetector (Fig. 6d). Alternatively, it could be possible to use the self-absorption of PVKs and the associated spectral change to determine the depth of interaction. With an accurate understanding of the optical properties of the crystal, the increased red shifting as a function of interaction depth would demonstrate the position of absorption, combined with a hyperspectral camera (Fig. 6e). Overall, there is a range of possibilities for the unique properties of PVKs to improve current PET systems, demonstrating the commercial potential of the material.

## 5. Conclusion

Owing to their intrinsic scintillation, combined with novel material properties, PVKs have the potential to increase the functionality of indirect detectors beyond current possibilities. The reported exceptional light yields and fast emission decay times holds promise for many applications, especially TOF systems, while the unique features of PVK materials expands the range of systems in which they can be utilised, from flexible systems to multispectral layered detectors. Furthermore, a low cost and simple fabrication may enable cost savings over existing materials, with the considerable simultaneous research on these materials for other optoelectronic applications relaxing the expected strain of upscaling to commercial levels. Despite admirable progress in a short time, there remains enormous room to develop these materials, primarily by reducing the disparity between experimental and maximum theoretical light yields, and the lack of application-specific degradation studies. Engineering materials with greater Stokes shifts, lower trap densities and/or with control of carrier density and recombination sites will bring improvements in photon outcoupling and luminescence yields; indeed, further advances in other PVK light emitting applications will feed back to further improve their scintillator applications. The promising radiation hardness and even self-healing already demonstrated in preliminary studies, combined with the carefully controlled end-use environments, means the use of halide perovskites as scintillator detectors will not be far from commercial application.

## Conflicts of interest

There are no conflicts to declare.

## Supplementary Material

TC-009-D1TC01595H-s001
