# Supplementary material for: Halide perovskites scintillators: unique promise and current limitations
Source: J Mater Chem C Mater. 2021 Jun 1;9(35):11588–604. doi: 10.1039/d1tc01595h (PMC8444306; doi:10.1039/d1tc01595h)
Supplement: TC-009-D1TC01595H-s001 [file TC-009-D1TC01595H-s001.pdf]

Electronic Supplementary Material (ESI) for Journal of Materials Chemistry C.
This journal is © The Royal Society of Chemistry 2021

Supplementary Information

Supplementary Table 1: Key Ionising Radiation Detector Figures of Merit

**Stopping Power**	Denotes a material's efficiency to attenuate incident photons. Highly dependent on input energy, as well as material density and mass attenuation coefficient, and governs the thickness of material required.
**Light Yield**	The number of photons emitted by the scintillator for a set input energy. (photons / MeV)
**Response Speed**	Assesses the temporal response of the device and can be quantified by the decay time of the scintillator emission. (ns)
**Afterglow**	Measures the residual light emission intensity a set time after excitation. Often caused by trapping and detrapping of charges before reaching luminescent centres. and represented by the percent of total emission at a certain time after excitation. (% at 10 ms)
**Emission Spectra**	The spectrum of the scintillator emission. Ideally this is tuned to the most suitable photodetector for the application e.g. PMTs.
**Dose Linearity**	Represents the range in which the scintillator response is linear with incident radiation dose. Particularly important in imaging applications.
**Energy Linearity**	Represents the range in which scintillator response is linear with incident photon energy. Particularly important in photon integration modes for energy resolution.
**Stability**	Consistency in emission performance after irradiation as well as withstanding the mechanical and environmental stresses required in the application. Exact requirements are highly application specific.
**Spatial Resolution**	Measures the smallest object that can be resolved by an imager. The modulation transfer function (MTF) is used in radiology applications, which quantifies the level of detail in an object that is retained by the image.
**DQE**	Quantifies the imaging ability of a detector. It is more all-encompassing than MTF by containing the signal contrast as well as the noise of the system.
